# Separation and HPLC Characterization of Active Natural Steroids in a Standardized Extract from the *Serratula coronata* Herb with Antiseborrheic Dermatitis Activity

**DOI:** 10.3390/ijerph17186453

**Published:** 2020-09-04

**Authors:** Marta Napierała, Joanna Nawrot, Justyna Gornowicz-Porowska, Ewa Florek, Arletta Moroch, Zygmunt Adamski, Anna Kroma, Izabela Miechowicz, Gerard Nowak

**Affiliations:** 1 Laboratory of Environmental Research, Department of Toxicology, Poznan University of Medical Sciences, 30 Dojazd Street, 60-631 Poznan, Poland; martan@ump.edu.pl (M.N.); eflorek@ump.edu.pl (E.F.); 2 Department of Medicinal and Cosmetic Natural Products, Poznan University of Medical Sciences, 33 Mazowiecka Street, 60-623 Poznan, Poland; joannac@ump.edu.pl (J.N.); arletam20@wp.pl (A.M.); kromkaania@interia.pl (A.K.); gnowak.gerard@gmail.com (G.N.); 3 Department of Dermatology, Poznan University of Medical Sciences, 49 Przybyszewskiego Street, 60-356 Poznan, Poland; adamskiz@poczta.onet.pl; 4 Department of Computer Science and Statistics, Poznan University of Medical Sciences, 79 Dabrowskiego Street, 60-529 Poznan, Poland; iza@ump.edu.pl

**Keywords:** phytoecdysteroids, *Serratula coronata*, seborrheic dermatitis

## Abstract

Phytoecdysteroids are natural compounds with therapeutic benefits in both humans and animals. The effectiveness of natural products with health potential is based on the activities and potencies of their active ingredients. In this study, dominant ecdysteroids—ajugasterone C, 20-hydroxyecdysone and polypodine B—from the *Serratula coronata* (*S. coronata*) herb were separated by column chromatography, identified by spectroscopic data and quantified by high-performance liquid chromatography with a diode array detector (HPLC-DAD). The obtained concentration of ecdysteroids (approximately 23%) in the *S. coronatae* herb extract enhances the possibility of their use in pharmaceutical and cosmetic products with high levels of phytoecdysteroids. Moreover, this study has shown a positive effect of ecdysteroids-containing cream on changes in quality of life and a beneficial effect in reducing the symptoms of seborrheic dermatitis. It has been demonstrated that the application of the cream with phytoecdysteroids resulted in a statistically significant alleviation of symptoms (*p* < 0.05), especially in terms of itching, pain or burning sensations in the affected areas in comparison to previous symptoms.

## 1. Introduction

Phytoecdysteroids are a class of chemical compounds (triterpenoids) that are synthesized by plants for defense against insects. Over 300 plant ecdysteroid analogues have been identified so far with distinctive chemical, physical and biological properties. These compounds are derivatives of cyclopentane perhydrophenantrene. The vast majority of them have 27 atoms of carbon, with a double bond between C7–C8 and a keto group on C6. Ecdysteroids are characterized by methyl groups on C10 and C13 in the ß configuration and by the double bond between C7–C8. Ecdysteroids isolated from the Centaureinae subtribe plants are further characterized by two OH groups on C2 and C3. Someecdysteroids—although only of vegetable origin—have an extra hydroxyl group on C1, C5, and C11 [1,2,3,4]. Plants usually contain a few major ecdysteroids, along with other closely related compounds that occur in lower amounts. The reliable qualitative and quantitative analysis of phytoecdysteroids isolated from plants are important for the development of new pharmaceutical products [2,3,4,5,6].

Pharmacological studies indicate numerous health benefits of phytoecdysteroids in both humans and animals. The compounds and materials in which they play a dominant role are very promising for phytotherapy [7,8,9,10]. Phytoecdysteroids have a positive effect on hypercholesterolemia, hyperglycemia, hypotension, infectious diseases and physical and mental weakness [8,11,12,13,14,15,16,17,18]. Puri et al. have found that 20-beta-hydroxyecdysone is efficient in the prevention of hot flushes in ovariectomized rats [19]. Several scientific reports have suggested the use of phytoecdysteroids as a supplement in the treatment of several diseases of the cardiovascular system [11,12,13,14,15,17]. A positive effect of phytoecdysteroids on human skin, including wound healing activity, has been postulated.

Seborrheic dermatitis (SD) is a common, chronic, recurrent inflammatory dermatosis that significantly decreases the quality of life of patients. Clinically, it is characterized by erythema and skin flaking, which occur most often in areas with a high concentration of sebaceous glands, including the face, scalp, ears, chest and body folds. This disease is a pervasive skin disorder that affects 3% of the population and may occur in patients from infancy to old age [20,21]. SD may appear as exfoliating yellow epidermis on a reddish background, sometimes accompanied by itching. Pruritus is often present in adult SD, especially when the scalp is involved. Due to pruritus and its chronic and recurring character, as well as the clinical manifestation in highly visible places, SD has a negative influence on patients’ psychical condition and their self-esteem [22].

According to the current knowledge on SD treatment, there is a lack of a single radical therapy that is 100% effective and results in permanent clinical outcomes [23,24]. The therapy is centered on the topical application of antifungals and anti-inflammatory agents [24]. The primary treatment is based on imidazole preparations and corticosteroids, which have little effect. The overall treatment is used only in severe SD cases and includes imidazole derivatives [25]. Therefore, SD still remains difficult to treat and requires a personalized approach for each patient.

In light of the above, there is an increasing interest in phytoecdysones—natural steroids found in plants—which are seen as an alternative treatment and/or complementary skin care treatment for SD [26]. However, their immunological significance and possible therapeutic use in SD still remain unclear. Thus, in this study, we focused particularly on the use of main phytoecdysteroids isolated from the *Serratula coronate* herb for skin disorders such as SD.

The aim of this study was to develop and validate a simple, sensitive and precise method for the isolation and quantification of dominant ecdysteroids—ajugasterone C (**1**), polypodine B (**2**) and 20-hydroxyecdysone (**3**)—from the *S. coronata* herb (Table 1). The next step was to assess the clinical potential of a cream with a 2% concentration of ecdysteroids for reducing the symptoms of SD.

## 2. Material and Methods

### 2.1. Plant Material

Herbs of *Serratula coronata* L. (Compositae/Asteraceae) were collected during the dry seasons of 2013–2016 (in May each year) from the Botanical Garden at the Department of Medicinal and Cosmetic Natural Products, University of Medical Sciences in Poznan (Poland), where the voucher specimens (No. 64/2009) are deposited. *S. coronata* seedswere provided by the Botanical Garden in Vacratot (Hungary). They were gathered from a natural habitat in the north of Hungary. The aerial parts and seeds of *S. coronata* were identified by Karol Latowski of Adam Mickiewicz University in Poznan.

### 2.2. Extractionand Identification of Ecdysteroids

Dried herbs of *S*. *coronata* (330 g) were crushed and soaked in methanol (MeOH; Merck KGaA, Darmstadt, Germany) (1.32 L, three times). The MeOH extract was evaporated, and the residue was dissolved in distilled water (0.5 L). The aqueous extract was shaken with ethyl acetate (AcOEt; Merck KGaA, Darmstadt, Germany) (three times with 0.5 L). The AcOEt extract was evaporated, leaving 7.35 g of residue.

The compounds were separated by column chromatography on silica gel with a size of 0.063–200 nm (Merck Art. 7733, Darmstadt, Germany). Selected fractions were further rechromatographed on silica gel with a size of <0.063 nm (Merck Art. 7729, Darmstadt, Germany).

The AcOEt extract was chromatographed on silica gel with mixtures of n-hexane and Me_2_CO (ratio 2:1) as eluent (Merck KGaA, Darmstadt, Germany). Two collected fractions needed purifying and were rechromatographed on silica gel with a mixture of dichloromethane (CH_2_Cl_2_; Merck KGaA, Darmstadt, Germany) and MeOH (ratio 5:1).

The ^1^H (600 MHz) and ^13^C NMR (150 MHz) spectra were run on a Bruker Avance 600 instrument (Bruker, Billerica, Massachusetts, US) in deuterated chloroform (CDCl_3_), deuterated methanol (CD_3_OD) or deuterated dimethyl sulfoxide (DMSO-*d_6_*). Chemical shifts are given in ppm (δ) and coupling constants in Hz (*J*). Melting points were determined on a Büchi B-540 Melting Point apparatus (BÜCHI Labortechnik AG, Flawil, Switzerland) and are uncorrected.

Dried and crushed herbs of *S. coronata* (620 g) was soaked three times in ethanol (EtOH) (2.85 L) for 24 h. The EtOH extract was evaporated and gave a residue—*Extractum siccum* (14 g)—which was used as the material for quantitative analysis and for the preparation of the standardized cream for application.

### 2.3. Ecdysteroids Characterization

For the characterization of compounds, ^1^H NMR analysis was performed. Signals of vegetable ecdysteroids have been seen in the proton spectra of all three analysed compounds (Table S1): double bonds between C7–C8 (characteristic doublets from 5.80 for compounds **1** and **3** to 5.85 for compound **2**).

The following signals are typical of compound **1** (Figure S1): a 4.10 ppm signal on C11, which is from the proton on the hydroxyl group; and a 1.58 ppm signal on C25, which means that there is no OH group in this location in the ecdysone’s aliphatic chain, resulting in typical signals of two methyl groups on C26 (0.916 ppm) and on C27 (0.920 ppm). The 2.38 ppm signal, on the other hand, indicating the presence of a proton on C5 in compound **3**’s spectrum, distinguishes it from compound **2** (Figures S2 and S3).

### 2.4. Quantification of Ecdysteroids Using HPLC-DAD Analysis

Ajugasterone C (**1**) and polypodine B (**2**) were used for analysis after their purification and identification by spectral methods. The ecdysteroid 20-Hydroxyecdysone (**3**) (1 mg/mL of methanol) in the form of a Certified Reference Material was purchased from Merck (KGaA, Germany). Solvents used for chromatographic analysis were of HPLC grade (Sigma Aldrich Sp. z o.o., Poznan, Poland).

The dry ethanol extract of the *Serratula coronata* herb was dissolved in methanol, in a final concentration of 20 g/L.

HPLC analyses were carried out using an Agilent 1200 SL system (Perlan Technologies, Germany). An analytical column with C8 silicone filling—5 μm (particle size), 125 × 4 mm (length × ext. diameter; LiChrospher^®^ 60 RP-select B, 5 µm, LiChroCART^®^ 125-4, Merck, Germany)—connected with a guard column with C8 silicone filling—5 μm, 4 × 4 mm (LiChrospher^®^ 60 RP-select B, 5 µm, LiChroCART^®^ 4-4, Merck, Germany)—was used to separate the compounds. HPLC analyses were carried out at ambient temperature (approximately 23 °C), at a 1 mL/min flow rate of the mobile phase, in UV absorption spectra, with a λ_max_ value of 242 nm in methanol, which is characteristic for ecdysteroids. Each sample injection of 15 µL was performed in triplicate. The analyses were carried out using a gradient elution of water and methanol. The gradient elution scheme was as follows: t_0_ (min), 5% MeOH; t_12_, 30% MeOH; t_25_, 30% MeOH; t_30_, 5% MeOH; with a post time of 5 min.Data and chromatograms were collected using Chem Station for LC 3D system Rev. B.04.01 SP1 software from Agilent Technologies (Waldbronn, Germany).

Quantitative analysis was performed using a standard external method. Stock solutions of the analyzed compounds (1.0 g/L of methanol) were used to prepare the calibration curves. The peak area ratios of compounds **1**–**3** were determined for each calibration sample using a calibration curve from 300 to 1000 mg/L. The linear equation describing the relationship between ajugasterone C (**1**), polypodine B (**2**) and 20-hydroxyecdysone (**3**) concentrations and peak area ratios was determined by least-squares weighted regression methods.The validation of the method was performed in accordance with the Food and Drug Administration (FDA) guidelines [27].Three standard curves were prepared on threedifferent days, and the appropriate regression statistics were determined. The limit of detection (LOD) andthe limit of quantitation (LOQ) were calculated by the determination of the signal-to-noise ratio (S/N) (LOD = 3 × (S/N); LOQ = 10 × (S/N)). For the intra-day assay, six replicates at three different concentrations of quality control (QC) samples—low (300 mg/L), middle (500 mg/L) and high (1000 mg/L)—were analyzed. For the inter-day assay, six replicates of low, middle and high-quality control samples were analyzed daily on three consecutive days. The mean, standard deviation, relative standard deviation (RSD) and accuracy of the intra-day and inter-day experiments were calculated. The accuracy was calculated on the basis of the given formula (mean concentration found/concentration taken) × 100. Accuracy and precision data for intra and inter-day assays for analyzed samples are given in Table 2.

### 2.5. Study Group and Antiseborrheic Activities

The study protocol was approved by the Bioethics Committee, based on Polish legislation and Good Clinical Practice at the Poznan University of Medical Sciences, Poznan, Poland (ethical approval number 1082/08). Written informed consent was obtained from all participants.

After dermatological consultation, the study included 36 patients: 17 women and 19 men aged 18–65. All of them were in a good general state of health. The patients were diagnosed with erythema and skin exfoliation and complained of itching. The changes in their skin were diagnosed as mild-to-moderate SD and were present on average for seven years. There were 64 diagnosed changes in total, located mostly (in 97%) on nasal alar creases, ears, central chest, anterior hairline.

The recipe for the cream was developed by the Department of Medicinal and Cosmetic Natural Products, Poznan University of Medical Sciences (Poznan, Poland). The active substance of the cream was previously described as a dry ethanol extract of the *Serratula coronata* herb containing three dominant ecdysteroids—ajugasterone C (**1**), polypodine B (**2**) and 20-hydroxyecdysone (**3**)—which constituted 2% of the cream. In this study, the examined ointments were prepared on the basis of a creamy Lekobaza^®^ Pharma Cosmetic base (Fagron, Kraków, Poland) with amphiphilic properties. Lekobaza is a commercially available, multi-component medium with a pH close to the skin condition [28].

Before the application of cream, all patients were subjected to skin allergy tests using patches with hypoallergenic and water-resistant chambers (IQ Ultimate™ by Chemotechnique Diagnostics) filled with a 25 μL aqueous extract from the *Serratula coronata* herb and 2 mg of the cream.

Each patient used 8 mg of the cream, which was applied directly to the changed skintwo times a week for six weeks. The effectiveness of the cream was evaluated by a dermatologist during each control visit—that is, after two, four, and six weeks of application—and documented in a Termoquolity index questionnaire. Patients in the study group did not use any other preparations for the skin changes and were asked not to ingest any oral medicines or food which could have affected the changed skin.

The effect of using the tested cream on changes in quality of life was determined using a Termoquality index survey (four-item itch questionnaire) [29]. An assessment of four pruritus features was made, which is the most troublesome symptom of SD. Patients (19 men and 17 women) scored on a scale of 1 to 5 the extent (location, duration), severity and frequency of pruritus and its effect on sleep difficulties.

### 2.6. Data Processing and Statistical Analysis

Test results were analyzed statistically using Statistica 12 software (StatSoftPoland, Cracow, Poland) by StatSoft. Statistical significance was α = 0.05. A result was deemed statistically significant if *p* < α. The Wilcoxon signed-rank test was used to compare the results before and after the application of cream.

## 3. Results and Discussion

### 3.1. Isolation of Dominant Phytoecdysones from the S. coronata Herb

As a result of our experiment, two fractions were isolated, one of which contained a chromatographically homogenous compound: ajugasterone C (**1**) (65.3 mg, m.p. 210–214 °C).

The second collected fraction (890 mg) contained a mixture of two compounds (but without the previously isolated one), which required purifying and was rechromatographed on silica gel with a different mixture: dimethyl chloride (CH_2_Cl_2_): methanol (MeOH) (ratio 10:1 then 7:1 and 5:1). As a result two compounds were isolated: polypodine B (**2**) (93 mg, m.p. 225–227 °C) and 20-hydroxyecdysone (**3**) (220 mg, 243–244 °C).

### 3.2. Thin Layer Chromatography (TLC) of the Isolated Compounds

**Figure 1:**
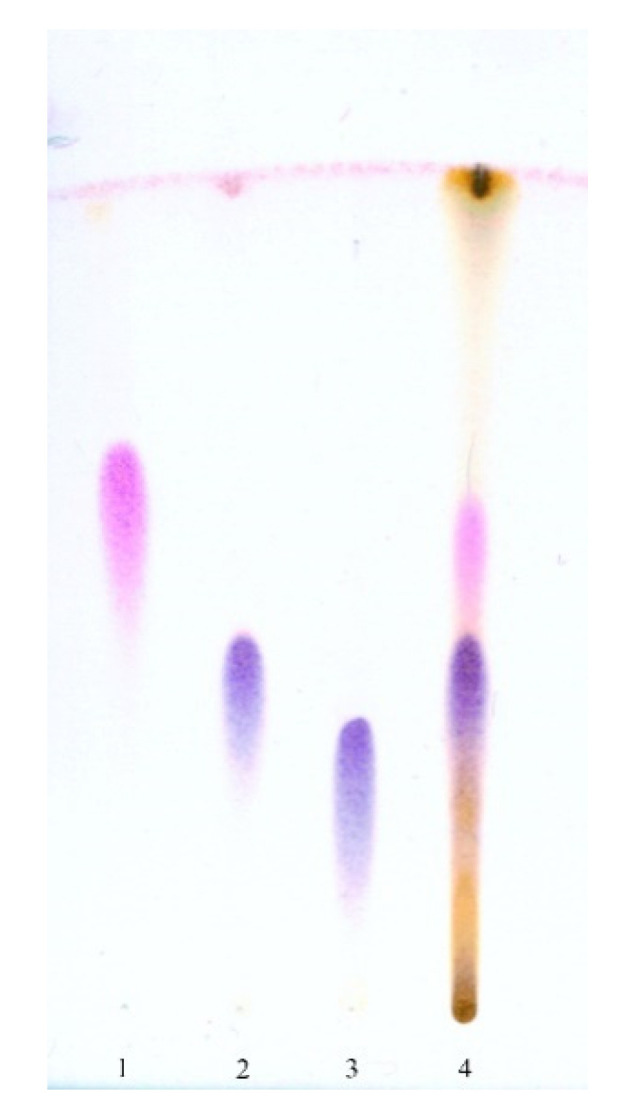
Thin-layer chromatography (TLC) of the purified AcOEt extract and phytoecdysteroids isolated from the *S. coronata* herb 1. ajugasterone C; 2. 20-hydroxyecdysone; 3. polypodine B; 4. AcOEt extract of the *S. coronata* herb; adsorbent: silica gel; mobile phase: n-hexane:Me2CO 2:1.

**Figure 2:**
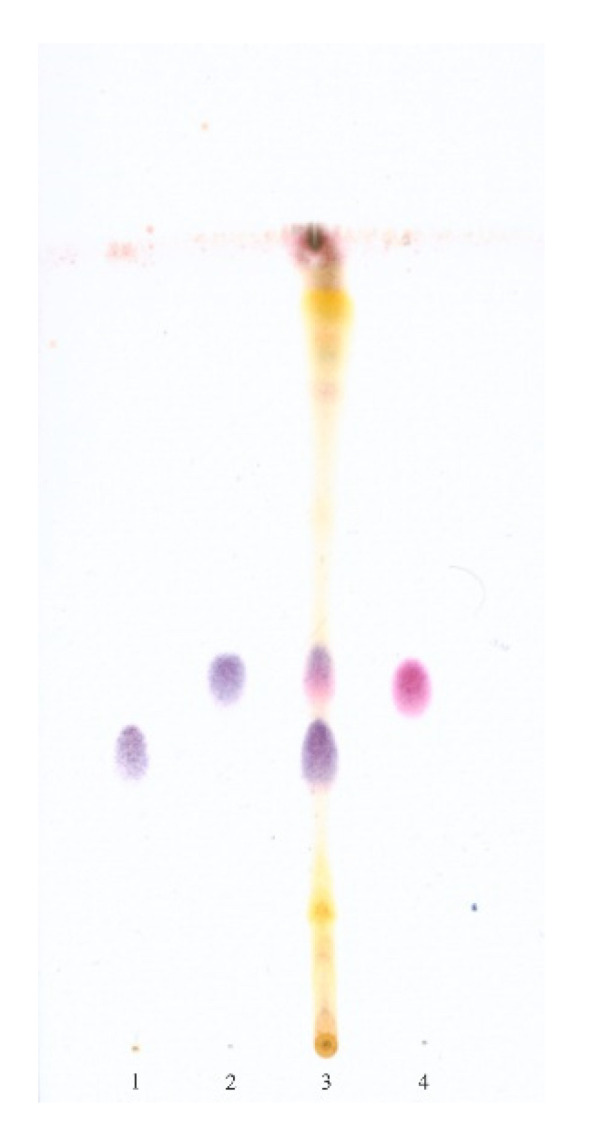
TLC of the purified AcOEt extract and phytoecdysteroids isolated from the *S. coronata* herb 1. 20-hydroxyecdysone; 2. polypodine B; 3. AcOEt extract of the *S. coronata* herb; 4. ajugasterone C; adsorbent: silica gel; mobile phase: CH2Cl2–MeOH 5:1.

After the visualization of the chromatograms of the AcOEt extract from the *S. coronata* herb, three principal spots appeared: one of them was purple and two were dark blue. Figure 1 shows a clear separation of the purple spot of compound **1**, and Figure 2 shows a clear separation between the two dark blue spots of compounds **2** and **3**. Interestingly, the TLC study showed the characteristic behaviour of polypodine B (**2**) on the chromatograms. This is a more polar compound than 20-hydroxyecdysone (**3**) (due to the additional hydroxyl group at C5) and should theoretically be placed below compound **3** on the chromatogram. However, this reasoning is valid only for a solvent system without methanol (Figure 1). When we used a developing phase containing methanol (Figure 2), compound **2** exhibited a higher running spot compared to compound **3**. A possible explanation of the change in the polarity of polypodine B (**2**) is the presence of both the hydroxyl group at position 5 and carbonyl oxygen at C6 [30].

### 3.3. HPLC Analysis

In this study, dominant ecdysteroids from *Serratula coronate* were successfully separated using the HPLC-DAD method. Calibration curves for all compounds were linear in the range of 300–1000 µg/mL (**1:** y = 12710x + 44.872, R^2^ = 0.9997; **2:** y = 16907x + 54.195, R^2^ = 0.9996; **3:** y = 11443x − 50.195, R^2^ = 0.9995).

The limits of detection (LOD) for compounds **1**–**3** were 18, 4.5 and 15 µg/mL, respectively. The limits of quantification (LOQ) for ajugasterone C (**1**), polypodine B (**2**), 20-hydroxyecdysone (**3**) were 60, 15 and 50 µg/mL, respectively.

The analysis of independent low, middle and high-quality control samples was used to perform the intra-day and inter-day assay. The intra-day RSD for compounds **1–3** ranged from 0.67% to 1.26% for ajugasterone C (**1**), from 0.60% to 0.95% for polypodine B (**2**) and from 0.51% to 1.10% for 20-hydroxyecdysone (**3**); additionally, an accuracy of 97% was achieved for compound **1**, between 98% and 106% for compound **2** and 96% and 102% for compound **3** (Table 2).

The inter-day relative standard deviation (RSD) for compounds **1**–**3** varied from 0.98% to 1.34% for **1**, from 0.72% to 0.93% for **2** and from 0.51% to 0.82% for **3**; furthermore, an accuracy of 96% was achieved for for ajugasterone C (**1**), between 98% and 106% for polypodine B (**2**) and 96% and 102% for 20-hydroxyecdysone (**3**) (Table 2). The above results indicated that the method was reliable, reproducible and accurate.

**Figure 3:**
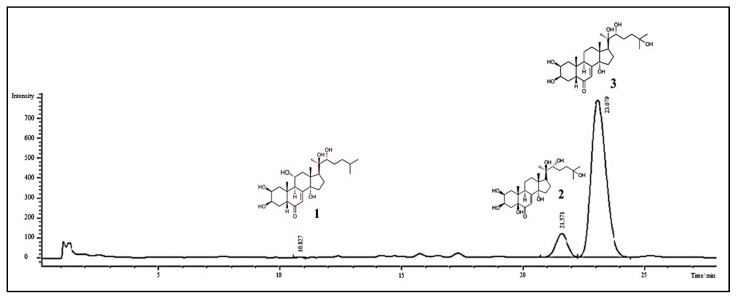
Chromatogram of *S. coronata* extract (10 mg/mL): ajugasterone C (**1**):less than limit of quantification (LOQ); polypodine B (**2**): 0.358 mg/mL (35.8 mg/g of extract); 20-hydroxyecdysone (**3**): 1.901 mg/mL (190.1 mg/g of extract); total compounds: 2.289 mg/mL (228.9 mg/g of extract) ≅ 22.59%.

The validated method was applied to the determination of compounds **1**–**3** in plant material extract (Figure 3) after column chromatography separation.

The obtained 22.89% concentration of phytoecdysteroids in the *S. coronata* herb extract of these compounds guarantees the possible use of the standardized extract as an effective ingredient in cosmetics and drug production with this unique group of natural compounds.

It should be noticed that isomers in rare cases are not identifiable by mass spectrometry (MS) because they produce identical spectra. In our work, the ^1^H NMR analysis indicated the details of the structures of the three compounds from the plant extract. Two of the compounds (ajugasterone C and 20-hydroxyecdysone) are isomers; therefore, by MS, the substitution sites ofOH groups are indistinguishable. The consequence of this approach is the misinterpretation of ajugasterone C and 20-hydroxyecdysone as synonyms. Therefore, we decided to use NMR in our study as the best method for structural analysis.

### 3.4. Examination of 2% Phytoecdysteroids Cream for Seborrheic Dermatitis

**Figure 4:**
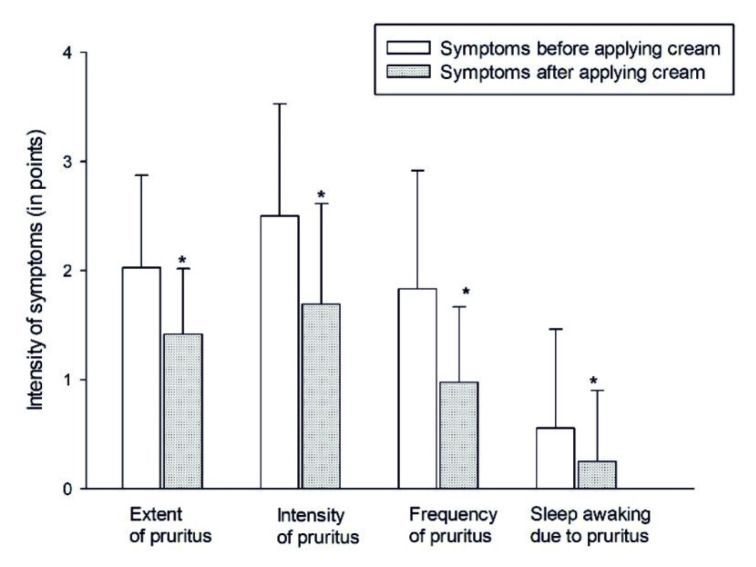
Comparison of termoquality index survey results before and after the usage of cream with 2% of ecdysteroids extract from the *S. coronata* herb. *—statistically significant difference compared to the result observed previously (*p* < 0.05; the Wilcoxon signed-rank test).

During the six weeks of the application of the cream, the quality of the patients’ life improved significantly, and in 97% of cases, following the use of the cream with phytoecdysteroids, the epidermis reverted to its physiological state as assessed by a dermatologist. Moreover, there was no report of any side effects of the cream. It has been shown that applying the cream with phytoecdysteroids resulted in a statistically significant alleviation of symptoms (*p* < 0.05), especially in terms of itching, pain or burning sensations in the affected areas in comparison to previous symptoms. The results obtained before and after the application of the cream are presented in Figure 4.

There are scientific reports which have suggested that phytoecdysteroids have anti-inflammatory and antioxidant properties as well as a positive effect on metabolic diseases, infectious diseases and physical and mental weakness [3,8,11,12,14,15,16,17,18,31]. Rosselli et al. have shown that ecdysones have cytotoxicity potential against rat C6 glioma cells [32]. However, further studies of the application of phytoecdysteroids in medicine are still needed.

The method used in this study could be adapted for the future evaluation of ajugasterone C (**1**), 20-hydroxyecdysone (**2**) and polypodine B (**3**) concentrations in patients’ plasma during the application of the cream. The obtained concentration of ecdysteroids (approximately 23%)in the *Serratula coronata* herb extract enhances their potential use in cosmetics and drug production with this unique group of natural compounds.

The results obtained in this study, after six weeks of usage of phytoecdysteroid-containing cream, were satisfactory. The quality of patients’ life improved considerably due to significant alleviation of symptoms (*p* < 0.05), especially in terms of itching, pain or burning sensations in the SD-affected areas. In 97% of the cases, following the use of the cream with phytoecdysteroids, the epidermis reverted to its physiological state. Moreover, there was no report of any side effects of the cream.

The precise molecular mechanisms describing the pharmacological activity of phytoecdysteroids in an unusual skin condition still remain unexplored. However, it is known that the action of phytoecdysteroids is associated with skin barrier function recovery. Data in the literature indicated that phytoecdysones might promote keratinocyte differentiation in vitro [33]. Thus, the positive effect of the application of phytoecdysteroid-containing cream in SD patients may be associated with the normalization of the keratinocytes differentiation process. It is postulated [34,35] that ecdysones may have a significant impact on the reduction of inflammation, probably due to their immunomodulatory function and the modulation of the level of the pro-inflammatory cytokine (e.g.,interleukin-6 (IL-6), tumor necrosis factor alpha (TNF-α)). Moreover, it has been reported that phytoecdysteroids improve skin quality by accelerating the healing of wounds and burns [36,37].Several studies [38] have claimed associations between *Malassezia restricta* lipase and SD. In light of this, ecdysteroids, especially 20-hydroxyecdysone, probably enhance antifungal immunity, resulting in a reduction of disease symptoms [39].

Therefore, it can be expected that the use of phytoecdysteroids might have a long-term impact on skin changes in patients with SD, and potential recurrences of the condition might be delayed. Cream with natural steroids may also be helpful for other skin disorders resulting in itching, as well as atopic dermatitis. No side effects were observed, which frequently occur during the application of a cream with synthetic steroids [23].

The results of our study give dermatologists, cosmetologists and patients hope for an efficient and safe skin care method. The positive results of applying the cream with a 2% content of ecdysteroids in seborrheic dermatitis are promising for the development of a new drug for skin diseases which cause pruritus.

## 4. Conclusions

The use of the *S. coronata* herb, with a high concentration of ecdysteroids—ajugasterone C (**1**), polypodine B(**2**) and 20-hydroxyecdysone (**3**)—and with bioactive properties, appears to be a promising strategy for the preparation of valuable pharmaceutical and cosmetic products. The positive results of applying the cream with a 2% standardized extract content of ecdysteroids in seborrheic dermatitis suggest its potential new application for skin diseases which cause pruritus. The simple but effective method for isolating active phytoecdysones presented in this paper maybe useful infuture study.

## Figures and Tables

**Table 1:** The chemical structures of dominant phytoecdysteroids from the *S. coronata* herb.

Source	Active Molecule/Ecdysteroids	Structure
***Serratula coronata* Herb**	Ajugasterone C (**1**)	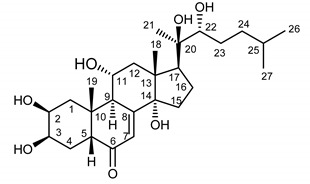
	Polypodine B (**2**)	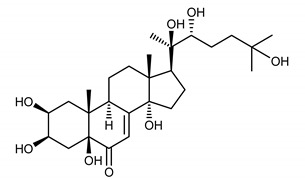
	20-hydroxyecdysone (**3**)	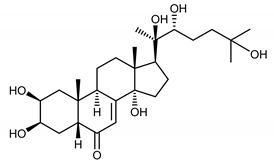

**Table 2:** Intra-day and inter-day precision and accuracy of compounds **1**–**3** (quality control, QC; relative standard deviation, RSD).

Nominal Concentration of QC Samples (µg/mL)	Predicted Concentration, Mean ± SD (µg/mL)	RSD (%)	Accuracy (%)
**A**. Intra-day assays; *n* = 6			
Ajugasterone C			
QC low 300	291 ± 1.95	0.67	97
QC middle 500	483 ± 3.39	0.70	97
QC high 1000	967 ± 12.23	1.26	97
Polypodine B			
QC low 300	318 ± 1.91	0.60	106
QC middle 500	491 ± 3.69	0.75	98
QC high 1000	997 ± 9.50	0.95	100
20-hydroxyecdysone			
QC low 300	288 ± 1.61	0.57	96
QC middle 500	510 ± 3.40	0.51	102
QC high 1000	991 ± 7.58	1.10	99
**B**. Inter-day assays; *n* = 6			
Ajugasterone C			
QC low 300	287 ± 3.02	1.05	96
QC middle 500	481 ± 4.71	0.98	96
QC high 1000	962 ± 12.85	1.34	96
Polypodine B			
QC low 300	317 ± 2.29	0.72	106
QC middle 500	489 ± 3.78	0.77	98
QC high 1000	995 ± 9.21	0.93	100
20-hydroxyecdysone			
QC low 300	287 ± 1.46	0.51	96
QC middle 500	511 ± 2.62	0.51	102
QC high 1000	991 ± 8.15	0.82	99

## Supplementary Materials

The following are available online at https://www.mdpi.com/1660-4601/17/18/6453/s1, Figure S1: ^1^H NMR (600 MHz, CD_3_OD) spectrum of compound **1**, Figure S2: ^1^H NMR (600 MHz, CD_3_OD) spectrum of compound **2**, Figure S3: ^1^H NMR (600 MHz, CD_3_OD) spectrum of compound **3**, Table S1: ^1^H NMR data (600.20 MHz) of ajugasterone C (**1**), polypodine B (**2**) and 20-hydroxyecdysone (**3**) (in CD_3_OD).
