# Supplementary material for: Separation and HPLC Characterization of Active Natural Steroids in a Standardized Extract from the Serratula coronata Herb with Antiseborrheic Dermatitis Activity
Source: Int J Environ Res Public Health. 2020 Sep 4;17(18):6453. doi: 10.3390/ijerph17186453 (PMC7557367; doi:10.3390/ijerph17186453)
Supplement: Supplementary file 1 [file ijerph-17-06453-s001.pdf]

SUPPLEMENTARY MATERIALS

Separation and HPLC Characterization of Active Natural Steroids in Standardized Extract from *Serratula coronata* Herb with Antiseborrheic Dermatitis activity

Marta Napierała [1], Joanna Nawrot [2], Justyna Gornowicz-Porowska [2,*], Ewa Florek [1], Arletta Moroch [2], Zygmunt Adamski [3], Anna Kroma [2], Izabela Miechowicz [4] and Gerard Nowak [2]

[1] Laboratory of Environmental Research, Department of Toxicology, Poznan University of Medical Sciences, 30 Dojazd Street, 60-617 Poznan, Poland
[2] Department of Medicinal and Cosmetic Natural Products, Poznan University of Medical Sciences, 33 Mazowiecka Street, 60-623 Poznan, Poland
[3] Department of Dermatology, Poznan University of Medical Sciences, 49 Przybyszewskiego Street, 60-356 Poznan, Poland
[4] Department of Computer Science and Statistics, Poznan University of Medical Sciences, 79 Dabrowskiego Street, 60-529 Poznan, Poland
* Correspondence: justynagornowicz1@poczta.onet.pl; Tel +48618691367

Figure S1. ^{1}H NMR (600 MHz, CD_3OD) spectrum of compound **1** (AJUGASTERONE C)

Figure S2. ^{1}H NMR (600 MHz, CD_3OD) spectrum of compound **2** (POLYPODINE B)

Figure S3. ^{1}H NMR (600 MHz, CD_3OD) spectrum of compound **3** (20-HYDROXYECDYSONE)

Table S1. ^{1}H NMR data (600,20 MHz) of ajugasterone C (**1**), polypodine B (**2**) and 20-hydroxyecdysone (**3**) (in CD_3OD)

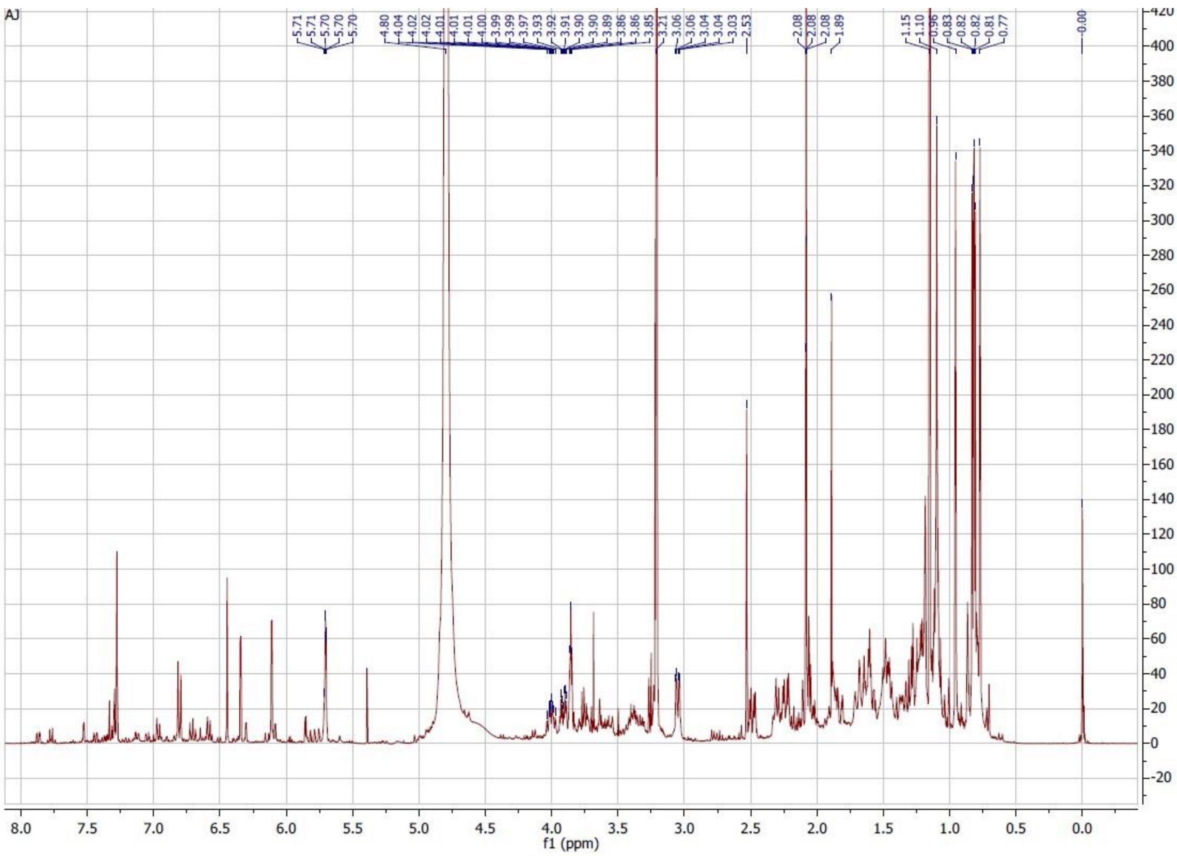

Figure S1. ^{1}H NMR (600 MHz, CD_3OD) spectrum of compound **1**.

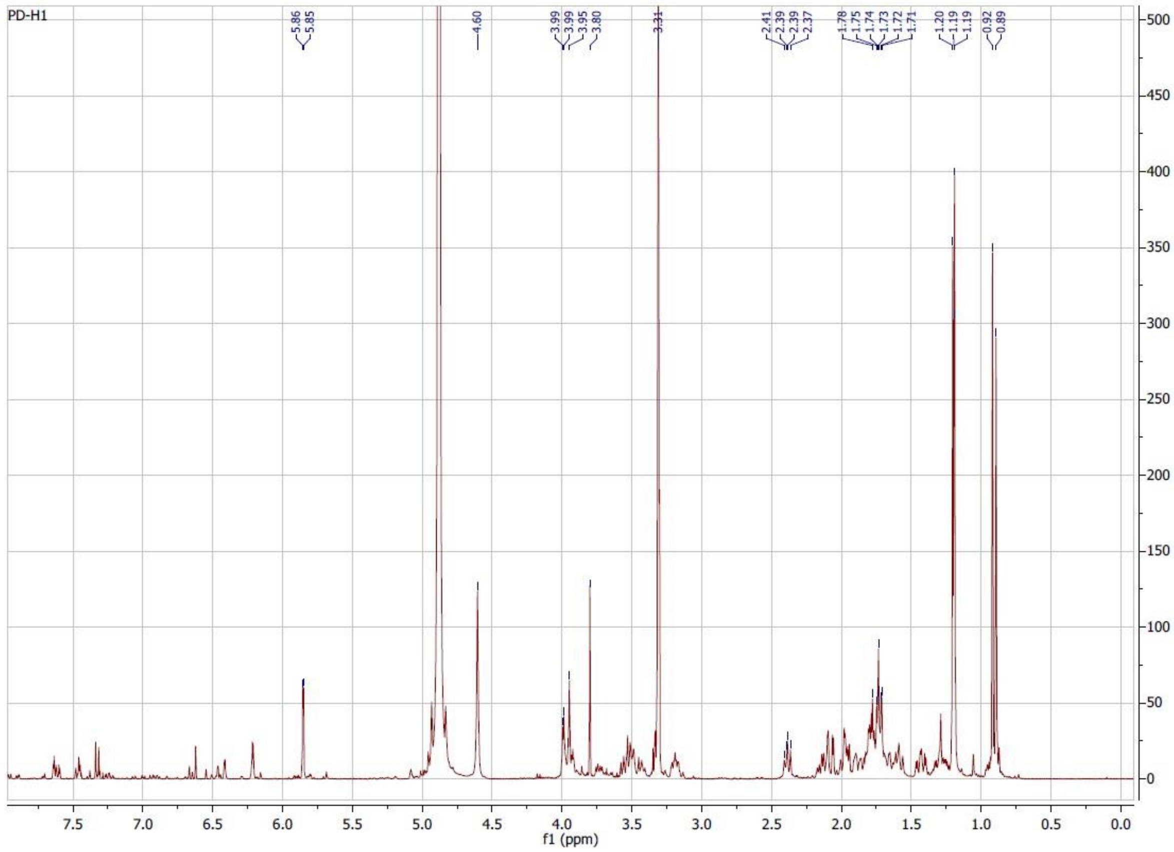

Figure S2. ^{1}H NMR (600 MHz, CD_3OD) spectrum of compound **2**.

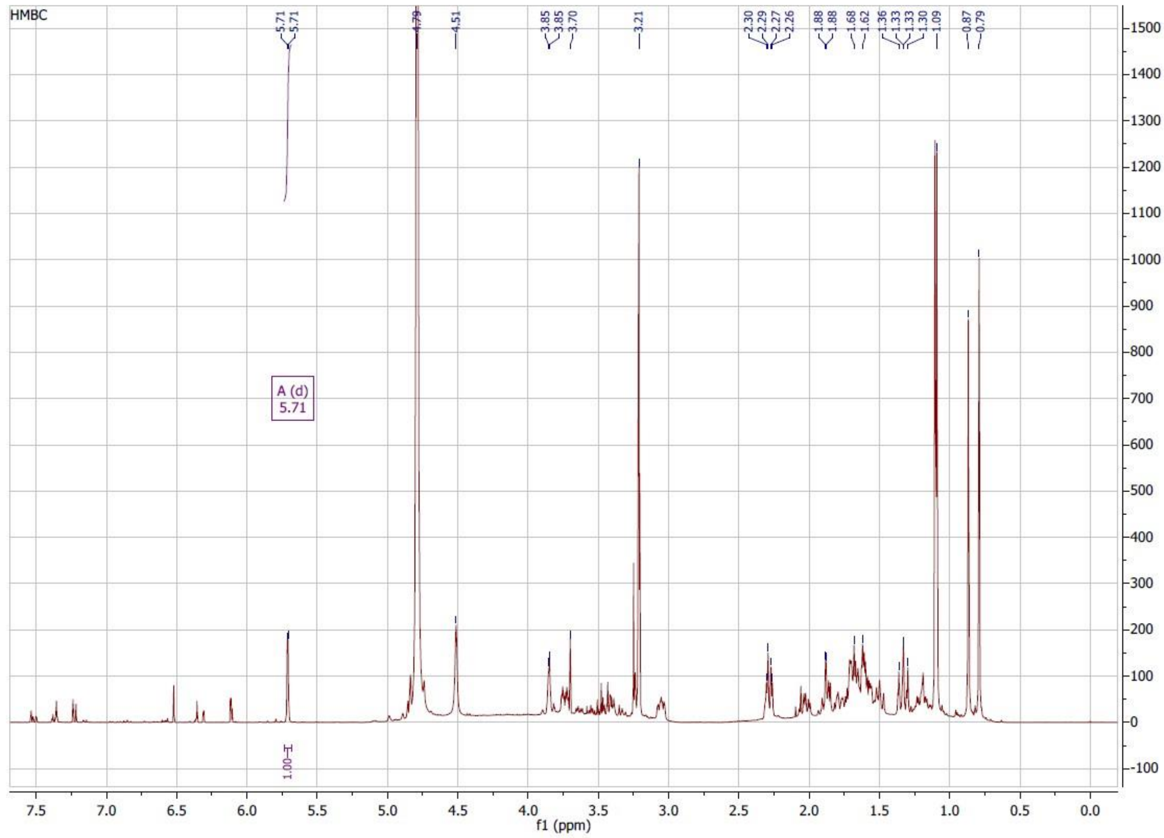

Figure S3. ^{1}H NMR (600 MHz, CD_3OD) spectrum of compound **3**.

Table S1. ^{1}H NMR data (600,20 MHz) of ajugasterone C (**1**), polypodine B (**2**) and 20-hydroxyecdysone (**3**) (in CD_3OD).

Proton	1✾✾✾H (ppm) (*J* Hz)	2✾✾✾H (ppm) (*J* Hz)	3✾✾H (ppm) (*J* Hz)
1a	2.58 dd (12.9; 4.0)	1.68 m	1.43 m
1b	1.38 m	1.78 m	1.78 dd (4.6; 13.3)
2	4.01 dt (4.0)	3.94 h (3.5; 6.5; 10.1)	3.83 tt (3.8; 8.2; 8.0)
3	3.95 m	3.99 bq (3.0; 6.2; 9.18)	3.94 bd (2.1)
4a	1.78 m	1.75 m	1.65 m
4b	1.69 m	2.07 dd (2.9; 14.8)	1.75 m
5	2.33 dd (3.7; 13.15)	-	2.38 dd (3.9; 10.0)
7	5.80 d (2.0)	5.85 d (2.7)	5.80 d (2.5)
9	3.15 m	3.19 m	3.14 dd (4.93)
11a	4.10 m (13.3)	1.72 m	1.65 m
11b	-	1.81 m	1.78 m
12a	2.21 m	2.13 m (4.9; 13.0)	2.13 ddd (4.8; 13.0;13.0)
12b	2.15 dd (5.9; 12.1)	1.88 m	-
15a	1.97 m	1.59 m	2.00 m
15b	1.56 m	-	1.55 m
16a	1.70 m	2.00 m	1.95 m
16b	1.99 m	1.74 m	1.75 m
17	2.41 m	2.39 m	2.39 m (3.9)
18	0.87 s	0.89 s	0.89 s
19	1.05 s	0.92 s	0.96 s
21	1.19 s	1.192 s	1.187 s
22	3.30 m	1.76 bm	3.33 d (1.5)
23a	1.54 m	1.28 m	1.30 m
23b	1.20 m	1.67 m	1.65 m
24a	1.47 m	1.77 m	1.75 m
24b	1.23 m	1.44 m	1.45 m
25	1.58 m	-	-
26	0.916 d (6.2)	1.187 s	1.195 s
27	0.920 d (6.2)	1.200 s	1.200 s
